# Assessment of Vitamin D Status in Great Apes in Human Care

**DOI:** 10.1002/zoo.21908

**Published:** 2025-06-10

**Authors:** Matyas Liptovszky, Christopher Reeves, Rachel Jarvis, Kerstin Baiker, Phillipa Dobbs, Kate White, Sophie Moittié

**Affiliations:** ^1^ School of Veterinary Medicine and Science University of Nottingham Sutton Bonington UK; ^2^ Department of Clinical Biochemistry Manchester University NHS Foundation Trust Manchester UK; ^3^ Veterinary Pathology Group Bristol UK; ^4^ Twycross Zoo, East Midland Zoological Society Atherstone UK; ^5^ School of Veterinary Medicine St George's University University Centre Grenada

**Keywords:** bonobo, chimpanzee, gorilla, great apes, orangutan, vitamin D

## Abstract

Reliably assessing vitamin D status in nonhuman great apes presents unique challenges, including the optimal collection, handling and storage of appropriate samples, assay selection, and interpretation of results. In recent decades, significant scientific evidence accumulated on these matters in humans, but a comprehensive overview of this topic in nonhuman great apes is currently lacking. This paper provides a review of the various sample types, storage and transport considerations, the wide range of available assays and their respective advantages and disadvantages, as well as important considerations for the reporting and interpretation of results, including environmental and individual animal‐related factors. A thorough discussion of the reasons behind inter‐ and intra‐assay variability of vitamin D metabolite concentration measurement is provided with the intent to support those caring for great apes to be able to reliably assess vitamin D status and interpret results. We also highlight the limitations of current human reference intervals, cover the existing literature on nonhuman great apes, and the importance of standardization across institutions to improve animal welfare and facilitate robust research. Finally, we provide a set of recommendations based on primarily current human literature to support zoo and sanctuary practitioners.

## Graphical abstract

Wild great apes, like these chimpanzees in Uganda, do seek direct sunshine even in their natural habitats.

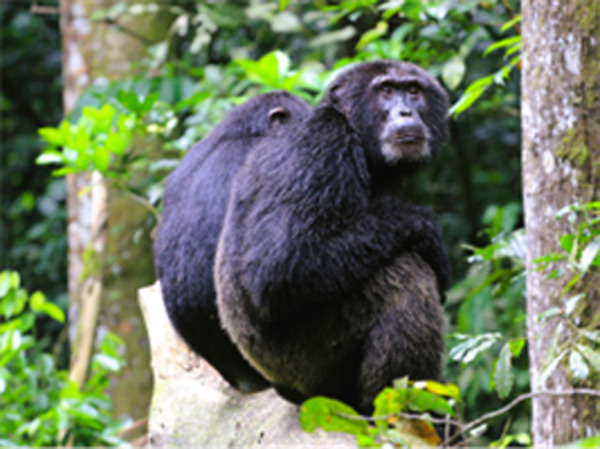

## 1 Introduction

Vitamin D plays a critical role in maintaining overall health across various species, including humans and nonhuman great apes (chimpanzees, bonobos, gorillas, and orangutans; *Hominidae*; further in this paper: great apes). Once primarily associated with calcium homeostasis and bone health, vitamin D has emerged as a potential modulator of various chronic diseases. Current research suggests it may play a role in reducing the risk of autoimmune disorders, cancer, respiratory and cardiovascular diseases (Holick 2009; Holick 2017). Understanding vitamin D metabolism, options for clinical monitoring of vitamin D status, and its impact on health are crucial for ensuring the welfare of great apes in human care.

Vitamin D deficiency in humans is described as a global health concern, with numerous large‐scale studies reporting low vitamin D status in most countries around the world, including those with high average ultraviolet (UV) B levels (Bouillon 2020; Mendes et al. 2020; Holick 2017). In humans, even short periods (e.g., 10–25 min/day in the United Kingdom) of exposure to unfiltered sunlight might elicit enough vitamin D production in the skin to reach minimum recommended concentrations (Webb 2018a, 2018b). However, vitamin D synthesis in the skin is affected by multiple factors including latitude, season, skin tone, age, and diseases; thus, achieving optimal vitamin D status might be challenging for some, depending on these factors (Rhodes et al. 2010; Tsiaras and Weinstock 2011).

Previous research also demonstrated that, similarly to humans, vitamin D status in great apes in zoos tends to be low, shows seasonal variations, and is significantly influenced by access (or the lack thereof) to unfiltered sunshine via outdoor access (Crissey 1999; Videan et al. 2007; Moittié et al. 2022; Bartlett et al. 2017). Clinical rickets is rare in great apes, but has been described in the literature (Junge 2009). Akin to humans, the negative impacts of vitamin D deficiency are poorly understood in these species. It may well be the case, for example, that vitamin D deficiency contributes to the development of idiopathic myocardial fibrosis, the progressive heart condition most commonly seen in captive great apes, though its pathogenesis is likely multifactorial (Baiker et al. 2018; Strong et al. 2020; Bartlett et al. 2017).

Compared to the human literature, reports on vitamin D status in nonhuman great apes are scarce. There is currently no data published on vitamin D status in any free‐ranging great apes. One study analyzed data from chimpanzees kept in African sanctuaries, demonstrating that chimpanzees living in sunnier habitats had significantly higher vitamin D blood levels, as assessed by measurement of 25‐hydroxyvitamin D (25(OH)D), than those living in densely vegetated tropical forest enclosures. This study also found that males, on average, had a significantly lower serum concentration than females, and juveniles had significantly lower concentrations than adults (Feltrer‐Rambaud et al. 2023). Studies from great apes outside their natural range in North American zoos had low 25(OH)D serum levels (mean 25(OH)D serum concentration ranging 13.1–16.7 ng/mL, 32.75–41.75 nmol/L) compared to humans. Great apes, and within those, chimpanzees, had the lowest levels of all nine primate species analyzed, despite dietary vitamin D provision exceeding the recommended levels (Crissey 1999). Similarly, we also found European chimpanzees to have low vitamin D serum levels in another study, with 1 in 3 analyzed samples demonstrating inadequate 25(OH)D levels using the human reference interval (sufficient over 50 nmol/L). The season of the year, health status of the animal, and unlimited outdoor access were all found to be significant factors in determining vitamin D status (Moittié et al. 2022). This mirrors the finding of an earlier study, in which chimpanzees were shown to experience vitamin D deficiency when housed without regular access to unfiltered sunlight, with effects being more acute for adult females (Videan et al. 2007). Similar findings were also demonstrated in Western lowland gorillas. Animals with almost daily outdoor access had significantly higher 25(OH)D blood levels than those managed primarily indoors. However, many individuals, even with outdoor access, had levels below the human recommendation, despite oral vitamin D supplementation (Bartlett et al. 2017).

There is an increased need for monitoring vitamin D status in great apes in human care, as most of these animals, even those kept in range country sanctuaries, might experience significantly lower levels of UVB exposure compared to their free‐living wild counterparts. Despite this, publications on this topic remain scarce, and there is no species‐specific reference interval established for any Hominoids, except humans. To further complicate progress on this matter, previous studies on great apes provided data that are likely not comparable between the studies, or with previous human research. Sample collection, storage, and handling, as well as assay selection, have not been standardized, and results are reported in different formats.

Standardization of the assessment of vitamin D status is challenging for several reasons. Pre‐analytical, as well as analytical factors can significantly affect the results, including amongst others, sample origin (e.g., age, sex, reproductive and health status), sampling time (e.g., time of the year), nutritional and environmental factors, sample type, assay selection, assay standardization and quality assurance. Results reporting (i.e., units of measurement) and interpretation (i.e., reference intervals) can complicate the matter post‐analytically (Altieri et al. 2020; Holmes et al. 2013; Holick 2009; Makris et al. 2021). There have been significant efforts made in human clinical and research laboratory settings to standardize some of these factors and improve the reliability of vitamin D status assessments in humans (Binkley et al. 2017; Cashman et al. 2013; Durazo‐Arvizu et al. 2017; Wise et al. 2021). There has been no similar effort so far in clinical veterinary medicine, likely because vitamin D assessment has not been widely used in most production and companion animal species to date. This results in poor comparability of the vitamin D research performed so far on great apes, and confusion as to how these results should be interpreted.

The aims of this paper are to

1. Review the existing human and veterinary literature on the assessment of vitamin D status as relevant for great apes in human care.
2. Propose a standardized approach for vitamin D status assessment, including sample collection, handling, vitamin D measurement, and reporting, in great apes based on best practices informed by relevant human and animal studies.

### 1.1 Measurement of Vitamin D

#### 1.1.1 Analyte Selection

A wide range of vitamin D metabolites have been identified since the discovery of vitamin D. Despite this, there is consensus that the most reliable predictor of vitamin D status is currently total 25‐hydroxyvitamin D (25(OH)D), which is the sum of 25(OH)D_2_ (originating from orally absorbed sources of vitamin D_2_) and 25(OH)D_3_ (originating either from orally absorbed sources of vitamin D_3_ or via sun exposure). 25(OH)D has a half‐life of 2–3 weeks in humans and reflects vitamin D both absorbed from food, as well as produced via skin exposure to UV. Modern assays are capable of distinguishing between 25(OH)D_2_ and 25(OH)D_3 (_Giustina et al. 2024; Holick 2009; Holick et al. 2011; Makris et al. 2021; Herrmann 2023). Research in both humans and great apes, however, demonstrates that 25(OH)D_2_ concentrations are generally low, compared to 25(OH)D_3_, partly due to differences in their metabolism (Makris et al. 2021; Moittié et al. 2022).

Most of the circulating 25(OH)D in humans is bound by the vitamin D binding protein (DBP) and albumin, with free 25(OH)D concentrations being less than 1% of the total. Under normal circumstances, free and total 25(OH)D concentrations are well correlated, and there is no clinical need to measure free 25(OH)D in most cases. This might not be true, however, in conditions which affect DBP, including pregnancy and acute illness (Giustina et al. 2024).

While 1,25‐(OH)_2_D is the key biologically active compound, several factors make it unsuitable for assessing vitamin D status in general. 1,25‐(OH)_2_D in the serum has a very short half‐life of 5–6 h, and circulating levels are 1000 times lower than that of 25‐(OH)D. Its serum level is also very closely regulated, and animals that are vitamin D deficient can have normal or elevated levels of 1,25‐(OH)_2_D (Holick 2009). Therefore, 1,25‐(OH)_2_D, is only recommended to be used to further diagnose some specific diseases of calcium, phosphate, and vitamin D metabolism, including certain genetic mutations, in humans (Herrmann 2023). These have not been described in nonhuman great apes to date.

24,25(OH)_2_D, the first product in the catabolism of vitamin D, gained interest recently as a potentially useful biomarker of vitamin D metabolism. Its serum levels are lower than 25(OH)D, but higher than 1,25‐(OH)_2_D, and its half‐life is about 7 days in humans, making it a more suitable compound for laboratory testing. It is currently debated whether 24,25(OH)_2_D levels alone or as a ratio (24,25(OH)_2_D/25(OH)D) could be a better indicator of vitamin D status in humans, than 25(OH)D alone. However, the measurement of 24,25(OH)_2_D is challenging, and the methodology is not well standardized. It is currently recommended in humans for the diagnosis of loss‐of‐action mutations of the gene encoding CYP24A1, but further research is required to establish if it will be suitable as a better biomarker of generic vitamin D status (Makris et al. 2021).

#### 1.1.2 Sample Selection, Collection, and Handling

Serum and plasma (using either EDTA or heparin as anticoagulant) are currently the most common biological sample types used for 25(OH)D measurement in humans (Holick et al. 2011; Makris et al. 2021; Zerwekh 2008). If available, serum is the preferred medium, to reduce the potential confounding impact of anticoagulants on the vitamin D assay, though human studies found similar 25(OH)D concentrations in serum and plasma (Makris et al. 2021; Mena‐Bravo et al. 2015; van der Vorm et al. 2022). Other vitamin D metabolites, however, might have different concentrations when measured from serum versus plasma (Mena‐Bravo et al. 2015). Further to this, samples should be ideally collected without the use of serum clot activator or similar additives, as these have been demonstrated to interfere with certain vitamin D assays (Elder et al. 2009; Mena‐Bravo et al. 2015; Yu et al. 2016). In humans, 25(OH)D concentrations in serum obtained from venous vs capillary blood samples were also not directly comparable (Lai et al. 2010). Most published great ape studies to date utilized venous serum samples to measure 25(OH)D concentration. While capillary blood sampling would be a promising and minimally invasive sample collection route in great apes, further research is required to establish its comparability to the existing venous results.

Vitamin D is stable in serum, and several publications investigated the changes in 25(OH)D concentration over time, as well as due to freeze‐thaw cycles in human serum. Up to 72 h storage of whole blood at room temperature before serum separation, as well as up to 7 days storage of serum at 6°C, or up to four freeze‐thaw cycles resulted in changes less than the analytical assay's precision, and therefore, were deemed acceptable (Antoniucci et al. 2005; Wielders and Wijnberg 2009; Yu et al. 2010). Serum samples can be stored at −80°C (and even at −20°C) long‐term, and centrifugation temperature also has no meaningful impact on 25(OH)D (Agborsangaya* et al. 2009; Borai et al. 2020; Colak et al. 2013; Hayden et al. 2015). A recent study demonstrated that 25(OH)D_3_ concentrations remained stable for up to 60 days, regardless of the storage temperature between −80°C and +25°C, and up to five freeze‐thaw cycles (Mena‐Bravo et al. 2019). Other vitamin D metabolites might be more prone to storage or freeze‐thaw dependent concentration changes in serum or plasma samples, therefore, it is important that the above findings are interpreted carefully, considering the analyte in question (El‐Khoury and Wang 2012; Mena‐Bravo et al. 2019). Hemolytic, icteric, or lipemic serum samples might erroneously influence 25(OH)D concentrations (Agarwal et al. 2015; Lee et al. 2024).

Dried blood spot (DBS) samples are simple and practical for sample collection without the need for centrifugation, separation, or freezing. They also allow for smaller samples volumes and are widely used in humans and animals in certain scenarios. It has been demonstrated in humans that DBS samples can be used for vitamin D measurement in a reliable way; however, results cannot be compared to serum results directly (Eyles et al. 2009; Heath et al. 2014). A calibration model to convert values between the two sample types has also been developed in humans (Heath et al. 2014). One study has discussed DBS in chimpanzees to date, in which serum and DBS samples from 17 chimpanzees were analyzed for 25(OH)D_3_ and 25(OH)D_2_ using liquid chromatography‐tandem mass spectrometry at two accredited laboratories. While DBS analysis showed acceptable intra‐assay and inter‐assay precision (6% and 12.6%, respectively), wider limits of agreement and the presence of both constant and proportional bias compared to serum measurements suggest challenges in interpreting DBS results, particularly those near clinical decision points. This study also indicates that the DBS method is not interchangeable with serum in chimpanzees, highlighting the need for further validation of this technique (Moittié et al. 2020).

Saliva would be an ideal specimen for vitamin D measurement in great apes, as its collection is easily facilitated in conscious animals via training, is noninvasive and can be repeated as frequently as needed. Despite this, to date there have been no studies investigating the utility of this biological sample in great apes. Vitamin D was measured from human saliva over 35 years ago (Fairney and Saphier 1987), but creating an assay that demonstrates a reliable correlation with serum levels remained challenging. Saliva flow rates can change throughout the day, and the complex nature of saliva itself as a matrix also poses challenges in the laboratory analysis. Human studies using this sample matrix also pointed out that 25(OH)D concentrations in saliva are generally 1000‐fold smaller than in serum (Alexandridou and Volmer 2022; Clarke et al. 2019; Higashi et al. 2008). It remains to be seen whether a suitably reliable assay can be developed utilizing saliva samples in great apes.

There are many other alternative sample types that are known to contain vitamin D, and potentially could be used in vitamin D status assessments, including various tissue types, hair, urine, and other body fluids (e.g., tear and milk). These could either provide easier, noninvasive access to samples, reveal longer‐term vitamin D status compared to serum, or support our understanding of vitamin D metabolism overall. However, each sample presents unique analytical challenges, therefore methods for sample preparation, extraction and analysis need to be developed and validated for each, before their use (Alexandridou and Volmer 2022). 25(OH)D levels measured in these alternative sample types do not necessarily correlate with serum concentrations, and currently, there is a lack of understanding of what would be considered normal for each of these. As an example, a study in young humans found no correlation between serum and hair 25(OH)D concentrations, and no effect of sun exposure or vitamin D supplementation on hair 25(OH)D3 concentrations (Gáll et al. 2022).

#### 1.1.3 Analytical Methods

25(OH)D concentrations can be measured by a range of methods, including competitive protein binding assay (CPBA), radioimmunoassay (RIA), enzyme immunoassay, high performance liquid chromatography, gas chromatography with mass spectrometry (GC‐MS), or liquid chromatography with tandem mass spectrometry (LC‐MS/MS), with the latter being currently considered the most accurate method (Lai et al. 2010; Makris et al. 2021; Herrmann 2023).

Due to the long‐standing challenges around defining vitamin D reference intervals, it is critical to understand how preanalytical and analytical variability can influence clinical decision making. These challenges have been well recognized for over 20 years, and considerable efforts have been made to mitigate the impact of variability (Binkley et al. 2004; Herrmann 2023). Nevertheless, various assays remain in use, and has been used for the analysis of historical samples, therefore a brief review of current and previous methods might be useful.

Manual or automated immunoassays are commonly used in laboratory practice to measure 25(OH)D concentration in serum or plasma. While many of these methods have been optimized over the last 20 years, considerable variability in analytical performance still exists, resulting in high inter‐ and intra‐assay variability. Assay bias of +/−20% is still relatively common, especially where the characteristics of the sample might be altered by biological processes, like pregnancy, kidney or liver disease (Makris et al. 2021; Herrmann 2023). Sample characteristics (including hemolysis, icterus, and lipemia) can also affect immunoassay results (Lee et al. 2024).

RIA based technology was developed in the mid‐1980s, and later became commercially available. This method is known to underestimate total 25(OH)D concentration in patients on vitamin D_2_ supplementation, was hard to automate, and was later mostly replaced by other methods which provide higher throughput (Lai et al. 2010).

Other methods previously used in laboratory practice included CPBA, which was developed in the 1970s. This method performed well, but required complex sample extraction and purification steps, which led to it being replaced by other technologies (Fairney et al. 1979; Lai et al. 2010). HPLC represented a significant progression from this method in the late 1970s, and is considered reliable, but requires expensive equipment, technical expertise, and large sample volume, as well as has limited throughput, and therefore, its use did not become widespread in clinical practice (Tripathi et al. 2022; Lai et al. 2010).

While GC‐MS methods are also described in the literature, these are not commonly used in laboratory practice, unlike LC‐MS/MS. LC‐MS/MS has excellent sensitivity on a wide concentration range. It is also very capable of separating different metabolites; therefore, can provide a reliable method for measuring 25(OH)D_2_ and 25(OH)D_3_, as well as potentially other vitamin D metabolites, from the same sample. LC‐MS/MS is currently considered the gold standard measurement method of 25(OH)D (Altieri et al. 2020). Despite their benefits, rapid, automated immunoassays also remain commonly used.

#### 1.1.4 Quality Assurance and Standardization

A critical challenge regarding measuring 25(OH)D concentration in biological samples, both in research and clinical settings, remains the large variability of measurements, due to pre‐analytical and analytical factors, many of which have already been outlined above. As a response to this issue, the standardization of 25(OH)D concentration measurement also received significant attention in recent years. The Vitamin D Standardization Program (VDSP) was established in 2010 for this purpose, and it provides a four‐step approach to achieve standardization. These include the creation of Reference Measure Systems (RMS), calibration of commercial assays to RMS, calibration of individual laboratory assays to RMS, and the verification of test performance (Binkley and Sempos 2014; Sempos et al. 2017). Several reference methods for 25(OH)D_2_ and 25(OH)D_3_ measurement with isotope dilution (ID) LC‐MS/MS have been developed and validated, acting as the gold standard RMPs for commercial and lab‐derived assays to be referenced against (Herrmann 2023). Building on this, the Centers for Disease Control (CDC, USA) started an international Vitamin D Standardization Certification Program, leading to an increased number of standardized 25(OH)D assays (Makris et al. 2021).

Quality assurance schemes specifically for laboratories measuring vitamin D metabolite concentration have also been implemented. These schemes distribute test samples to participating laboratories and ensure centrally coordinated comparison of results. At least two such schemes currently exist, run by the College of American Pathologists (CAP), and the Vitamin D External Quality Assessment Scheme (DEQAS), ensuring independent oversight of clinical and research laboratories and that measurements do not exceed predefined bias (Makris et al. 2021).

Finally, an important issue remains the use of data collected previously through non‐standardized assays. As outlined above, inter‐, and intra‐assay variability significantly contributed to the confusion around what is considered normal 25(OH)D concentration in humans, and this issue continues in animal studies. To address this issue, the VDSP has developed a methodology for the retrospective standardization of serum 25(OH)D data. This enables the comparison of data obtained through a non‐standardized method with a current standardized method, but it requires a certain number of samples being banked and re‐measured with the new method. The guidelines provide information in relation to sample size calculations, statistical theory to implement this conversion, as well as two options for the retrospective standardization protocols (Binkley et al. 2017; Durazo‐Arvizu et al. 2017).

### 1.2 Reporting and Interpreting Results

#### 1.2.1 Reporting

In view of the inter‐ and intra‐assay variability, it is crucial to ensure that any research reporting on vitamin D measurement describes the methods used in sufficient detail, including the standardization (or lack of) the used assay, so these issues can be reliably assessed (Giustina et al. 2024). Absolute 25(OH)D concentrations, however, should still be interpreted carefully, taking into consideration the sample type, and the standardization status of the used assay (Makris et al. 2021). Meta‐analyses should only report on studies which used standardized assays, or those where retrospective standardization using the VDSP methods have been undertaken (Makris et al. 2021).

A further challenge is that 25(OH)D concentrations are commonly reported using two different units in the literature: mass unit (ng/mL) and molar (SI) unit (nmol/L). The recommended unit of reporting is nmol/L, alternatively, both units should be reported (Giustina et al. 2024; Makris et al. 2021). While a simple conversion factor of 1 ng/mL = 2.5 nmol/L exists, attention must be paid when reporting and interpreting results to avoid any interpretation errors.

#### 1.2.2 Individual and Environmental Factors

There is currently no evidence that species, individual, or environmental factors should be considered in the way vitamin D metabolite concentrations are measured. However, these factors have significant implications for the interpretation of results. Further to this, it is important to note that in humans, pregnancy is associated with increased inter‐assay variability of 25(OH)D measurement, as immunoassays tend to underestimate 25(OH)D concentration in these samples, compared to LC‐MS/MS‐based assays. This is thought to be due to the increase in vitamin D binding protein (VDP), and the inability of immunoassays to completely dissociate 25(OH)D from the VDP (Makris et al. 2021).

The Vitamin D status of an individual is influenced by several factors. Of environmental factors, season of the year has the largest effect. Unfiltered sunlight (and its UVB component) is the main source of vitamin D, therefore exposure to this clearly influences the vitamin D status of the individual (Feltrer‐Rambaud et al. 2023). Great apes living in the Northern hemisphere, therefore, typically present a higher concentration of 25(OH)D during the summer and autumn (high UVB) period (Moittié et al. 2022). Even in chimpanzees living in an African sanctuary, sun exposure was found to be a significant predictor of 25(OH)D concentration (Feltrer‐Rambaud et al. 2023). As 25(OH)D has a half‐life of 2–3 weeks in humans, it is important to consider that the measured concentration shows a cumulative effect of UVB exposure, rather than reflecting dynamic, short‐term changes.

Factors that relate to the individual also influence vitamin D status, and these should be considered when interpreting laboratory results. These can include age, sex, body condition, time spent outdoors, genetic factors, as well as some diseases and pregnancy. Makris et al. provide a comprehensive review of these factors in humans, highlighting that age, body mass index, and sex have limited impact (Makris et al. 2021). Previous studies in humans demonstrated that cutaneous vitamin D production decreases with age, quantified as a 13% reduction per decade, though sun exposure remains the most effective source of vitamin D throughout life (Chalcraft et al. 2020). Skin color has a more important role, especially at more Northerly latitudes, as demonstrated by a 15–20‐fold higher prevalence of vitamin D deficiency in African Americans compared to European Americans (Ames et al. 2021; Darling et al. 2013; Webb 2018a). Time spent outdoors is, however, the most important factor, with 5–30 min of daily exposure to unfiltered sunlight to the naked arms or legs currently assumed to provide adequate vitamin D synthesis in humans (Makris et al. 2021; Rhodes et al. 2010). Time spent outdoors is important even in areas with high UVB irradiation levels, including in Spain and India (Dharmshaktu et al. 2019; Valtueña et al. 2014).

So far, there is limited research on these factors in nonhuman great apes, but previous research demonstrated similar findings in chimpanzees and gorillas, where outdoor access was a significant determinant of serum 25(OH)D concentration (Videan et al. 2007; Moittié et al. 2022; Bartlett et al. 2017). From individual cases, it also appears that skin pigmentation is an important factor as well, as the highest concentrations of serum 25(OH)D were measured in lightly colored chimpanzees (Strong et al. 2020). Health status (Moittié et al. 2022), and age (Feltrer‐Rambaud et al. 2023) have been found to significantly influence 25(OH)D in chimpanzees. It is currently unknown how sex, body condition, or pregnancy influences 25(OH)D concentrations in great apes. Caution should be taken in interpreting individual measurements, and if possible, repeated measurements over time should be used to assess the vitamin D status of an individual or group of great apes.

For obvious reasons, dietary vitamin D supplementation, regardless of whether vitamin D_2_ or D_3_, also has an impact on an individual's vitamin D status. Vitamin D supplementation in the general human population, with the broad goal of the prevention of vitamin D deficiency, remains controversial. While national guidelines exist, recommended supplementation levels, as well as target 25(OH)D concentrations differ (Bouillon et al. 2022; Giustina et al. 2024; Holick et al. 2011). Few studies investigated the impact of dietary vitamin D supplementation on 25(OH)D concentration in great apes; however, so far, no positive correlation was revealed in chimpanzees and gorillas (Moittié et al. 2022; Bartlett et al. 2017). It is worth noting that generally vitamin D_3_ and D_2_ are not considered equivalent when used for supplementation and vitamin D_3_ is considered superior in elevating serum 25(OH)D concentration, both in humans and nonhuman primates (Giustina et al. 2024; MARX et al. 1989). The bolus dosing of vitamin D_2_ in humans has also been linked to adverse health outcomes recently (Giustina et al. 2024). However, vitamin D_2_ supplementation has been successfully used to treat clinical rickets in juvenile chimpanzees (Junge 2009), and remains widely used in human medicine.

#### 1.2.3 Reference Intervals

What is considered “normal” or sufficient vitamin D status is currently debated in the human medical literature. Partly, this is caused by the large inter‐ and intra‐assay variability of 25(OH)D measurement methods as outlined above. However, other considerations also include whether the guidelines take a clinical or public health approach to develop recommendations, that is, whether the aim is to prevent an adverse clinical outcome in every individual, or to balance costs and benefits. Defining the cut‐off values based solely or mostly on providing protection from rickets and other musculoskeletal diseases, or whether a more holistic approach is taken, also influences the outcome. Some have even suggested that individually calculated, rather than fixed, reference intervals should be established, considering the pre‐analytical and analytical variability of measurement (Ferrari et al. 2017). This has created significant complexity around defining clinical cut‐off points to determine supplementation, as well as other interventions to prevent disease‐inducing vitamin D deficiency (Sempos and Binkley 2020).

Guidelines for the measurement and clinical use of vitamin D metabolites for humans have been and are being developed typically by national public health or nutrition agencies, as well as by various nongovernmental organizations. As the relevance of these for great apes is not clear, we provide two examples here: the Institute of Medicine (IoM) (Institute of Medicine 2011 and the Endocrine Society (Holick et al. 2011) guidelines. These are summarized in Table 1 and are markedly different in both what they consider vitamin D deficient, as well as sufficient. It is important to note, that while the Endocrine Society issued its guidelines as a clinical tool, the IoM later clarified that their guidelines are not for clinical use, and in its development they took a public health approach, meaning it does not aim to achieve disease prevention in every individual, but for about 97.5% of the population (Sempos and Binkley 2020).

**TABLE 1:** Comparison of guidelines on vitamin D deficiency, insufficiency, and sufficiency in humans.

Guidelines	Year	Deficient	Insufficient	Sufficient
Endocrine Society Clinical Practice Guidelines (Holick et al. 2011)	2011	< 50 nmol/L	52.5–72.5 nmol/L	> 75 nmol/L
Institute of Medicine Institute of Medicine 2011)	2011	< 30 nmol/L	30–50 nmol/L	> 50 nmol/L

Currently, no similar recommendations exist for nonhuman great apes, and reference intervals for these species are also lacking. The primary issue to develop reference intervals is access to free‐ranging wild animal samples in adequate numbers, as well as difficulty in undertaking analysis in the field using a standardized assay, or the export and transportation to undertake such analysis.

While there is currently no free‐ranging wild great ape data published, range country sanctuary chimpanzees have been used as a proxy to understand what normal vitamin D status could look like for this species in Africa. One study found a median serum 25(OH)D concentration of 46.24 nmol/L, with a very wide range of 17.1–109.23 nmol/L across individuals. Males had significantly lower average concentration than females, and infants also had significantly lower average concentration than adults. Sun exposure (determined by the type of habitat within the animals' enclosure) was also a significant determinant of serum 25(OH)D concentration (Feltrer‐Rambaud et al. 2023).

It is notable that these results are similar to those collected in European zoos, where a median of 57.7 nmol/L (range: 5–151 nmol/L) has been found, despite most samples being collected at latitudes above 46^◦^ North. This might be due to the inclusion of vitamin D in commercial pelleted diets in European zoos, as only 23 of the 245 samples were from animals taking further oral vitamin D supplements (Moittié et al. 2022). Further to this, a small number of chimpanzees (*n* = 14) housed in another African sanctuary were reported to have significantly higher serum 25(OH)D_3_ concentrations (118+/−47 nmol/L) (Janssens 2019).

It is therefore currently unknown what 25(OH)D concentration would be considered normal in great apes. One can assume the precautionary position to take the human guidelines as a starting point, and define vitamin D deficiency as serum levels under 30‐50 nmol/L, and sufficiency above 50–75 nmol/L. It is important to consider that over the UVB‐poor winter period, human serum 25(OH)D concentrations tend to decrease by 10–25 nmol/L, therefore, a higher cut‐off value might be prudent if measured at the end of summer to prevent deficiency over the winter. It has also been suggested that, due to the difficulties around ascertaining inter‐ and intra‐assay variability, it might be desirable clinically to aim for a higher value of 100 nmol/L, which would then in nearly every case guarantee a “real” 25(OH)D value of over 75 nmol/L (Holick et al. 2011). Given the large evidence base from human studies, as well as the more limited research on chimpanzees, it is well established that this concentration would be safe while maximizing health benefits.

## 2 Summary

Vitamin D deficiency remains a significant health concern in humans and nonhuman great apes. Organizations caring for these animals have a responsibility to provide optimal husbandry, nutrition, and care to achieve positive welfare outcomes. From human and animal studies, unlimited outdoor access appears to be a key component to this, which can help in preventing vitamin D deficiency as well as providing additional benefits.

To ensure the appropriate assessment of vitamin D status, and avoid the pitfalls of quantifying vitamin D metabolite concentrations in nonhuman great ape samples, we compiled the following recommendations based on current scientific evidence, as outlined in this paper:

1. Similar to humans, 25(OH)D should be used to assess vitamin D status in great apes.
2. Serum is the preferred sample and should be collected in plain tubes with no additives, using venipuncture, rather than capillary blood samples. If serum is not available, plasma can also be used with either EDTA or heparin as anticoagulant.
3. Serum samples can be centrifuged at room temperature and stored at +4°C if 25(OH)D measurement is possible within 72 h. Samples can be transported to a laboratory at ambient temperature or on ice packs. Dry ice shipment is not required for routine samples.
4. If laboratory measurement will take place after more than 72 h, serum samples can be stored frozen, ideally at −80°C, but short to medium term storage at −20°C also seems acceptable.
5. If direct comparison of measured values is a goal (e.g., research projects or assessment of vitamin D status in groups), samples should ideally be analyzed in batches, rather than individually, to minimize intra‐assay variability. More importantly, the same laboratory and assay should be used in these cases.
6. If available, LC‐MS/MS appears to provide the most reliable 25(OH)D concentration measurements currently. In the absence of this, standardized assays should be used, and 25(OH)D measurement should be undertaken in laboratories that participate in a quality assessment scheme (CAP or DEQAS) with satisfactory results.
7. Results should be reported using SI units (nmol/L), or both SI and mass units (ng/mL) together. The unit of measurements should be always checked before interpreting results.
8. Care should be taken when interpreting individual 25(OH)D results, including the consideration of environmental and individual related factors—latitude, season, age, health status, outdoor access, dietary vitamin D intake—as well as assay standardization.
9. Due to the lack of species‐specific reference intervals of 25(OH)D concentration, clinicians need to use their own judgment to interpret results as normal, insufficient, or deficient. Human guidelines tend to advocate for 50–75 nmol/L as the acceptable minimum for sufficiency, and some African sanctuary‐housed chimpanzees demonstrated even higher values.
10. Reference intervals should only be generated using established guidelines, an adequate sample size, and a standardized assay in a laboratory participating in an external quality assessment scheme.
11. If 25(OH)D measurements are taken for research purposes, especially if the assay is not currently standardized or the measurements are undertaken outside laboratories that participate in a quality assessment scheme (e.g., in the field), serum samples should be banked appropriately to enable retrospective standardization of results using the VDSP methodology.
12. Any publication reporting on vitamin D status of nonhuman great apes should provide appropriate signalment and relevant history of the animals (including husbandry and nutrition information which might influence 25(OH)D concentrations), as well as details of sample collection, processing, and laboratory methodology (including the assay used, its standardization status, and whether the laboratory is a participant of an external quality assessment scheme).

However, practitioners should contact the laboratory undertaking measurement before sample submission to ensure that the assay used is validated for the sample type, as well as to discuss sample size, storage, and transport requirements, as these might differ between different laboratories.

The above steps, supported by scientific evidence mostly derived from human studies, can help achieve a more standardized and comparable vitamin D status assessment in great apes. There remains, however, a wide range of matters that are not appropriately addressed in nonhuman great apes in relation to vitamin D status, including standardized recommendations for the screening and testing to establish vitamin D status, as well as husbandry and nutritional practices to prevent deficiencies.

Therefore, we propose to undertake a structured, iterative, expert elicitation process to develop consensus guidelines on the evaluation and prevention of vitamin D deficiency in nonhuman great apes.

## Ethics Statement

The authors have nothing to report.

## Conflicts of Interest

The authors declare no conflicts of interest.

## Data Availability

Data sharing is not applicable to this article as no datasets were generated or analyzed during the current study.
